# Profiling Personality to Predict Athletes’ Academic Achievement: Cross-Cultural Analysis

**DOI:** 10.3390/bs16030461

**Published:** 2026-03-20

**Authors:** Aleksandra M. Rogowska, Cezary Kuśnierz, Iuliia Pavlova

**Affiliations:** 1 Institute of Psychology, University of Opole, 45-040 Opole, Poland; 2 Faculty of Physical Education and Physiotherapy, Opole University of Technology, 45-758 Opole, Poland; c.kusnierz@po.edu.pl; 3 Department of Theory and Methods of Physical Culture, Ivan Boberskyj Lviv State University of Physical Culture, 79007 Lviv, Ukraine; pavlova.j.o@gmail.com

**Keywords:** academic achievement, athlete academic performance, athlete personality types, Big Five traits of student athletes, cross-cultural sport psychology, grade point average (GPA), latent profile analysis (LPA), personality, sex

## Abstract

Research using latent profile analysis (LPA) has yielded inconsistent results regarding the number of personality profiles among athletes, the specific configuration of the Big Five traits, and their interpretation. This study seeks to explore personality types by excluding additional variables from the LPA model, aiming to assess how well personality profiles are universal (independent of gender and cultural context) and can predict academic achievement in student athletes. A cross-sectional study was conducted using a paper-and-pencil questionnaire among 424 student athletes from two universities in Poland and Ukraine. The average age of participants was 20 years old (*M* = 20.01; *SD* = 2.48), 62% were male, 53% lived in Poland, and 58% studied Sports Sciences vs. 42% Physical Education. The Mini-International Personality Item Pool (Mini-IPIP) was used to assess the Big Five personality traits, and grade point average (GPA) was used to measure students’ academic achievements in the last semester. The LPA identified four personality profiles: (1) *Restrained Neurotic* (Profile 1, 32%), *Open Extravert* (Profile 2, 42%), *Competitive Neurotic* (Profile 3, 17%), and *Cooperative Perfectionist* (Profile 4, 8%). Profiles 1, 3, and 4 showed similarly low levels of emotional stability, extraversion, and intellect but differed significantly in agreeableness and conscientiousness. Gender and country differences across athletes representing specific profiles were also noted. Profile 2 showed the strongest link with academic achievement. Hierarchical multiple linear regression showed that LPA profiles explained only 2% of GPA variance, compared to Big Five personality traits (9%) and demographic variables, such as sex, country, and study major (8%), which were also included in the following steps in the regression model, explaining only 9% and 8%, respectively. Most student athletes (52%) with personality profiles 1 (*Restrained Neurotic*), 3 (*Competitive Neurotic*), and 4 (*Cooperative Perfectionist*) may require psychological training to better cope with negative emotions and stress arising in competitive and academic settings. Profile 2 (*Open Extravert*) seems to be the most adaptive and potentially successful personality type. Personality types are, at least to some extent, related to gender and country of residence. More cross-cultural research is required to further verify the types of athletic personalities.

## 1. Introduction

### 1.1. Personality Traits Among Athletic Students

Academic achievements among student athletes influence educational progression, career opportunities, and well-being during early adulthood. Academic success is measured by grade point average (GPA). The relationship between sports participation and academic outcomes is complex. Research shows that sports participation correlates with equal or better academic outcomes when institutions support dual-career development (Gorry, 2016; Guo et al., 2019; Ishihara et al., 2020). However, academic challenges increase in high-intensity contexts, where training, competition, travel, time constraints, and fatigue can reduce study time (He et al., 2024; Pinto-Escalona et al., 2022). Cross-cultural differences in study requirements affect student athletes’ academic achievements, particularly in Eastern Europe, where athletes prepare during Physical Education (PE) studies to become PE teachers and coaches.

PE studies in Poland and Ukraine require strong sporting proficiency and thorough education (in pedagogy, methodology, dietetics, and self-regulation), leading to higher earnings. GPA assessment comprises grades in (1) practical sports training and (2) theoretical subjects related to sports theory and teaching methodology. The PE curriculum consists of 60% theoretical and 40% practical classes in the first two years. Practical classes focus on improving fitness and sports techniques. In the third and fourth years, practical classes increase to 60%, with greater emphasis on teaching methodology and on organizing physical activity. Academic success for PE students enables them to turn their passion for sports into a professional career as a personal trainer, sports instructor, sports manager, physiotherapist, or specialist in recreational centers. Success in academic sports allows athletes to combine athletic careers with professional development, increasing their chances of success after graduation and improving their well-being. Research on the factors that determine PE students’ academic achievement is crucial for designing a curriculum that supports their development and future success.

Academic achievements among sports sciences university students are affected by personality traits and sport-related factors, including sports commitment, athletic self-efficacy, and study approaches (Aykora & Dinçer, 2022; Şirin et al., 2023). Research shows conscientiousness is the strongest predictor across educational contexts (Bhattacharjee & RamKumar, 2025; Boonyapison et al., 2025; Mammadov, 2022; O’Connor & Paunonen, 2007; Stajkovic et al., 2018). A meta-analysis revealed that conscientiousness explains about 28% of the variance in academic achievement beyond cognitive ability (Stajkovic et al., 2018). Openness and agreeableness contribute positively to academic success, whereas neuroticism negatively affects it (Komarraju et al., 2011; Zell & Lesick, 2022). Extraversion shows mixed effects, and its influence may depend on the learning environment (Zell & Lesick, 2022).

Personality traits predict sports participation and performance, and targeted interventions considering these traits can enhance athletic outcomes (Li et al., 2024; Shuai et al., 2023). Traits such as extraversion, conscientiousness, and openness are positively associated with exercise motivation and athletic performance, whereas neuroticism shows negative associations (Shuai et al., 2023; Q. Yang et al., 2025; Zhang, 2025). Successful athletes score higher in agreeableness, conscientiousness, and emotional stability than less successful athletes, with individual-sport athletes showing greater energy and openness than team-sport athletes (Steca et al., 2018). Gender differences exist in emotional intelligence and exercise motivation, though Big Five scores often show no significant gender differences among sports students (Rehman et al., 2025; Q. Yang et al., 2025). Studies with varsity athletes reported no significant sex differences in Big Five traits (Piepiora, 2024; Rehman et al., 2025). However, male athletes tend to score higher on extraversion and emotional stability than females, potentially influencing well-being and performance (Solmaz et al., 2024). Female athletes show higher trait anxiety and emotional reactivity, while males exhibit higher physical aggressiveness (Kemarat et al., 2022; Obmiński et al., 2020). While core Big Five traits remain similar across genders among athletes, gender differences emerge in emotional and behavioral expressions, necessitating gender-sensitive psychological support in sports (Obmiński et al., 2020; Rehman et al., 2025; Solmaz et al., 2024).

### 1.2. A Person-Centered Approach to Examining Personality Among Athletes

Because academic achievement is influenced by complex, interacting variables rather than a single factor, a comprehensive approach is necessary to better support student athletes in their academic careers. In contrast to variable-centered methods that treat personality traits as separate factors influencing athletes’ academic success, we propose a person-centered approach. A person-centered approach with latent profile analysis (LPA) can help identify distinct subgroups (profiles) within a sample of sports sciences students, revealing complex patterns in personality traits that individual-level analysis cannot capture. In particular, this approach leads to more nuanced, practical insights for tailored interventions, for understanding heterogeneity, and for connecting personality theory to practice, as opposed to variable-centered methods that oversimplify by focusing on the average relationship (based on correlations). A person-centered approach better reflects the reality that personality operates as a system, rather than as a collection of independent traits. This allows for more precise predictions of how different trait configurations influence academic success rather than simply identifying individuals who are already struggling. Defining personality profiles enables the early identification of students who may be at risk of academic difficulties before apparent problems arise. LPA enables the implementation of preventive measures and the tailoring of psychological support to the specific needs of different personality types, rather than simply reacting to existing setbacks.

Among adolescents, the five personality types identified by LPA showed significant differences in physical activity levels, highlighting the predictive value of combined personality traits for exercise engagement (Chen et al., 2024). These types included the low-control conservative, balanced development, optimistic action, independent avoidance, and introverted vulnerable groups. In another study, Wang et al. (2024) identified five personality profiles in broader adult samples: moderate, reserved, confident, resilient, and vulnerable. These personality types were linked to exercise behavior, with resilient and confident profiles associated with greater engagement in physical activity. Research conducted among competitive climbers classified personality and temperament traits into four profiles, namely curious and impulsive, emotionally unstable, healthy, and measured and compliant, which differed significantly in sensation-seeking traits (Rumbold et al., 2021). It is important to note that impulsivity and sensation seeking were included in the LPA, alongside personality. Among young elite athletes, LPA identified three profiles based on two of the Big Five personality traits: neuroticism and conscientiousness. These profiles included maladaptive (high levels of conscientiousness and neuroticism), adaptive (high levels of conscientiousness and moderate levels of neuroticism), and highly adaptive (moderate levels of conscientiousness and low levels of neuroticism) types, showing distinct differences in stress appraisals and sleep quality among athletes (Nédélec et al., 2020).

### 1.3. Objectives of the Study

Previous variable-centered research has shown that athletes generally exhibit a different set of personality traits than non-athletes (e.g., lower levels of neuroticism and higher levels of extraversion). However, individual differences also exist among athletes by sport and gender. LPA can better explain this heterogeneity by identifying a set of traits common to distinct groups of athletes representing different sports disciplines. Student-athlete intervention or psychological support programs primarily focus on holistic development, addressing mental health, academic success, and athletic performance through specialized counseling, mentoring, and structured training. LPA approaches can enhance the effectiveness of these interventions by identifying specific, homogeneous subgroups of athletes based on their unique personality profiles, enabling highly personalized support rather than “one-size-fits-all.” Summarizing previous LPA studies, research has more intricately explained variations in athletic behavior, motivation, and performance, with conscientiousness and extraversion often linked to better outcomes. Overall, the person-centered approach appears highly beneficial for understanding how Big Five traits cluster among athletes and how they relate to their psychological and performance characteristics. However, the varying numbers and configurations of personality traits across studies suggest that further research is needed to clarify these differences. Significant differences in LPA results may arise from the inclusion of different variables in the analyses. Therefore, LPA studies based solely on the Big Five personality traits among athletes can identify their general personality profiles, independent of other variables. Additionally, cross-cultural differences may influence specific LPA results, necessitating further research.

This study aimed to examine personality profiles to predict athletes’ academic achievements. Understanding this issue is crucial for implementing interventions during academic years to help athletes achieve academic success, which has long-term consequences for their future sports careers and overall well-being. LPA will be performed on five personality traits that have been confirmed as universal across cultures, especially neuroticism, openness, and conscientiousness (Allik & McCrae, 2004; Rolland, 2002; Schmitt et al., 2007). LPA relies on the interaction of personality traits as a system, offering a richer understanding than methods that focus on isolated variables. This approach better reflects the complexity and heterogeneity of the real world, making the results more relevant and easier to apply in practice. The discovery of unique profiles allows the development of personalized intervention strategies. Personality profiles can help explain why athletes have different predispositions toward academic achievement, offering more profound insights into their behaviors and performance.

Based on previous research, the following hypotheses will be examined:

1. High scores in conscientiousness, intellect (openness), agreeableness, and emotional stability (low neuroticism) are associated with high academic achievement among athletes.
2. Personality profiles can predict academic achievement among athletes.

## 2. Materials and Methods

### 2.1. Study Design and Procedure

This cross-sectional study was conducted simultaneously in Poland and Ukraine during the second semester of the 2019/2020 academic year. The Ethics Committee of Scientific Research approved the study protocol at the University of Opole (No. 1/2020, dated 22 April 2020) and the Bioethics Committee of the Lviv State University of Physical Culture (No. LSUPC#2019-03-0903, dated 9 March 2019). The sample size for the analysis of variance (ANOVA) was determined a priori using G*Power software version 3.1.9.7. Assuming a medium effect size for a one-way ANOVA (*f* = 0.25), four groups for comparison, a minimal value of *p* = 0.05, and a power of 0.95, the expected sample size was *N* = 400 participants. There is no single, universal formula for sample size in LPA, as it depends on the degree of separation between profiles and the model complexity (Nylund-Gibson & Choi, 2018; Weller et al., 2020). However, a minimum sample size of 300–500 observations is typically used, with a minimum of 30–50 individuals per profile (or at least 5% of the total sample) to avoid estimation problems (Edelsbrunner et al., 2023; Mathew & Doorenbos, 2022; Wurpts & Geiser, 2014). Simulation studies suggest that a sample size of 300–1000 individuals is appropriate for achieving desirable power in LPA.

Recruitment used classroom-based convenience sampling in the Physical Education (PE) and Sports Sciences (SS) departments in both universities. During scheduled classes, the students were informed about the study and invited to participate via an in-class announcement. Participation was voluntary and anonymous. Students completed a paper-and-pencil questionnaire in their native language during class time, with the lecturer’s permission and without incentives for participation. The number of eligible students approached and the participation rate were not recorded systematically during data collection; therefore, the response/participation rates could not be reported.

Students from the PE and SS departments voluntarily and anonymously completed a paper-and-pencil standardized questionnaire in their native language during classes at the university, with the consent of the lecturers. The inclusion criterion was being a student of Physical Education (PE) or Sports Sciences (SS) and providing consent to participate in the research. The exclusion criterion was more than 5% missing data on Mini-IPIP trait scores and/or failure to provide GPA from the last semester. Exclusions were applied during data screening prior to the analyses; participants with incomplete data on variables used in a given analysis were removed listwise for that analysis. Initially, 473 students were invited to participate; 12 (2.5%) refused, and 37 (7.8%) were rejected because their questionnaires were invalid (missing data > 5%).

A total of 424 athletic students participated in the study, indicating an appropriate sample size for all statistical tests. In this study, “athletic students” were operationally defined as students enrolled in PE or SS programs who reported regular participation in sports training during the semester as part of their main sports discipline. Training exposure was quantified by self-reported weekly training volume; in the present sample, students reported an average of approximately 10 h of sports training per week across a range of individual and team disciplines, including swimming, athletics, team sports (e.g., football, handball, volleyball, and basketball), and individual sports (e.g., fitness, self-defense, aerobics, table tennis, and strength exercises).

This study used secondary data from a published article on the relationships among gender, personality, motivation, and academic achievement (Kuśnierz et al., 2020). The original article (Kuśnierz et al., 2020) examined the relationships between personality, academic motivation, and GPA using a variable-centered approach. A hierarchical multiple regression analysis was performed to examine predictors of academic achievement, including gender, country, three dimensions of motivation (intrinsic, extrinsic, and amotivation), and five personality traits (extraversion, emotional stability, intellect, agreeableness, and conscientiousness). In this study, only two variables (Big Five personality traits and GPA) were analyzed using a person-centered approach, which deepens previous findings and expands possible interpretations of the interaction between personality and academic achievement.

### 2.2. Participants’ Characteristics

The study involved 424 students majoring in Physical Education (PE) and Sports Sciences (SS), aged 18 to 29 (*M* = 20.01; *SD* = 2.48). Among them, 261 were male (62%), and 163 were female (38%), with 224 Polish (53%) and 200 Ukrainian (47%) athletes. Most participants were undergraduates (*n* = 342, 81%) and spanned all academic years from the first to the fifth. They specialized in two main areas: SS Coach or Instructor (*n* = 245, 58%) and PE Teacher (*n* = 179, 42%). On average, each student engaged in 10 h of sports training per week, focusing on their chosen discipline, including swimming, athletics, team sports (such as football, handball, volleyball, and basketball), and individual sports (such as fitness, self-defense, aerobics, table tennis, and strength-training exercises). A significant number of PE students are affiliated with the Academic Sports Association, allowing them to regularly participate in additional sports training and competitions in their free time.

### 2.3. Measures

#### 2.3.1. Personality

The Mini-International Personality Item Pool (Mini-IPIP) is a concise 20-item questionnaire designed to assess the Big Five personality factors (Donnellan et al., 2006). The Mini-IPIP captures the core aspects of the Big Five traits, enabling meaningful comparisons and profiling in large samples. The Mini-IPIP is widely used in research because of its brevity and demonstrated validity across diverse populations, including athletes. Its concise format reduces participant burden, which is crucial in athletic settings where time and motivation for long assessments may be limited. Each of the 20 items is rated on a five-point Likert scale, ranging from 1 (*Not at all accurate*) to 5 (*Very accurate*), and is distributed across five scales, each containing four items: emotional stability (ES; the inverse of neuroticism), extraversion (E), intellect (I; equivalent to openness), agreeableness (A), and conscientiousness (C). The Polish validation study of the Mini-IPIP showed acceptable Cronbach’s alpha coefficients of 0.70, 0.78, 0.65, 0.71, and 0.75 for ES, E, I, A, and C, respectively (Topolewska et al., 2014). The Ukrainian version was translated in accordance with the cultural adaptation guidelines (Wild et al., 2005, 2009). The reliability of these scales was measured in this study using Cronbach’s α coefficient, yielding values of 0.57, 0.69, 0.60, 0.54, and 0.67 in the total sample; 0.45, 0.56, 0.59, 0.49, and 0.64 in the Ukrainian sample; and 0.66, 0.79, 0.63, 0.60, and 0.70 in the Polish sample, for ES, E, I, A, and C, respectively.

#### 2.3.2. Academic Achievements

Self-reported general point average (GPA) was used to evaluate academic achievement. It was calculated as an average on a 6-point scale from all courses completed in the previous semester, in accordance with the Ukrainian and Polish grading systems. The scale ranges from 2.0 = *Insufficient* to 3.0 = *Sufficient*, 3.5 = *Satisfactory*, 4.0 = Good, 4.5 = *Very Good*, and 5.0 = *Excellent*. Higher values indicate better academic achievement. While there may be some institutional differences in GPA, the grading system is the same in both countries, using the same scale and assigning similar meanings to each grade. For example, a grade of 2 means the student failed the course and must repeat it the following year, whereas a grade of 5 is the highest and indicates excellent mastery of the course material. Therefore, the results of the Ukrainian and Polish student athletes can be compared.

### 2.4. Statistical Analysis

Several descriptive statistics were used for the preliminary assessment of the data, including the mean (*M*), standard deviation (*SD*), skewness, and kurtosis. Because the sample size was large (*N* > 200) and the skewness and kurtosis values did not exceed 1, parametric tests were conducted (George & Mallery, 2020). Partial eta-squared (η^2^*_p_*) was used to assess effect size in ANOVA, and a *p*-value of 0.05 (5%) was adopted as the acceptable level of significance. The associations between variables were assessed using Pearson’s *r*. The LPA for the five personality traits was assessed using the glca R package of the JAMOVI software, version. 2.6.44. The analysis started with five latent classes, and the final LPA was performed with four classes, yielding the best parameters among the options based on an analytic hierarchy process (AHP) (Akogul & Erisoglu, 2017). Combining model-based clustering with AHP leverages the strengths of both methods to improve the accuracy of determining the number of clusters in a dataset. The AHP was used to structure the decision-making process by considering multiple criteria (AIC, AWE, BIC, CLC, and KIC) and their relative importance, which was determined using a pairwise comparison matrix. This structured approach enables a more comprehensive evaluation of the information criteria, resulting in a more reliable determination of the optimal number of clusters. Using the AHP, the approach synthesizes information from different criteria. It prioritizes the decision rule that yields the highest composite relative importance vector (C-RIV), thereby selecting the best alternative for the number of clusters (Akogul & Erisoglu, 2017). In this study, model selection was guided by multiple considerations, including information criteria, classification quality, class proportions, and interpretability. Because fit indices can point to different solutions, we prioritized the model that balanced adequate fit with meaningful, stable class interpretation and non-trivial class sizes.

As part of the sensitivity analysis, the Pearson χ^2^ test of independence was used to compare the proportions of the four distinguished personality profiles between females and males. Multiple hierarchical linear regression was used to test the effect of LPA profiling on the academic achievements of athletes by controlling for sex (male = 1, female = 0), study specialization (PE Teacher = 1, Sports Coach or PA Instructor = 0), and country (Poland = 1, Ukraine = 0) in the first step and five personality traits in the second step. The LPA profiles were included in the analysis in the third step to examine which part of the variance in academic achievements could be explained separately by these LPA classes. All assumptions for the linear regression model were met, including linearity (based on scatter plot), normality (Kolmogorov–Smirnov statistic = 0.53, *p* = 0.192), independence of errors (autocorrelation −0.017, Durbin–Watson statistic 2.03, *p* = 0.836), normality of residuals (Q-Q plot), homoscedasticity (Goldfeld–Quandt statistic 0.53, *p* = 1.000), and no multicollinearity (Variance Inflation Factor ranged between 1.023 and 1.358, and tolerance ranged between 0.736 and 0.997). All analyses were performed using JAMOVI software version 2.6.44.

## 3. Results

### 3.1. Descriptive Statistics

Initially, we performed statistical analyses to examine the parametric characteristics of the variables, their distributions, and the bivariate relationships between them. Table 1 presents the descriptive statistics. Academic achievement shows a positive relationship with intellect, exhibiting a medium effect, and with conscientiousness and extraversion, both showing small effects. However, it is not related to emotional stability and agreeableness. Emotional stability is positively correlated with extraversion and intellect and negatively with agreeableness, with all correlations reflecting small effects. A high intellect score is linked to high extraversion (medium effect) and agreeableness (small effect). Additionally, there are positive but small associations between conscientiousness and agreeableness scores.

### 3.2. Latent Profile Analysis

An analytic hierarchy process (AHP), based on the fit indices Akaike’s Information Criterion (AIC), Bayesian Information Criterion (BIC), Kullback Information Criterion (KIC), Approximate Weight of Evidence (AWE), and Classification Likelihood Criterion (CLC), was used to select the best solution for LPA (Akogul & Erisoglu, 2017). AHP structures problems into hierarchies (goal, criteria, alternatives). The main aim is to find the “best” LPA model that fits the data while using fewer parameters, avoiding noise from numerous criteria. The AIC and KIC aim to select models that best predict outcomes while remaining simple (penalizing complex models). BIC and AWE penalize complex models more strongly than AIC, better finding the “true” model. The CLC focuses on the model’s classification of alternatives. The process compares different versions of the decision hierarchy and selects the model with the lowest indices, balancing data explanation and simplicity (Akogul & Erisoglu, 2017). In this study, AHP suggests that Model 1 with 4 classes is the best solution for LPA (Table 2).

**Figure 1:**
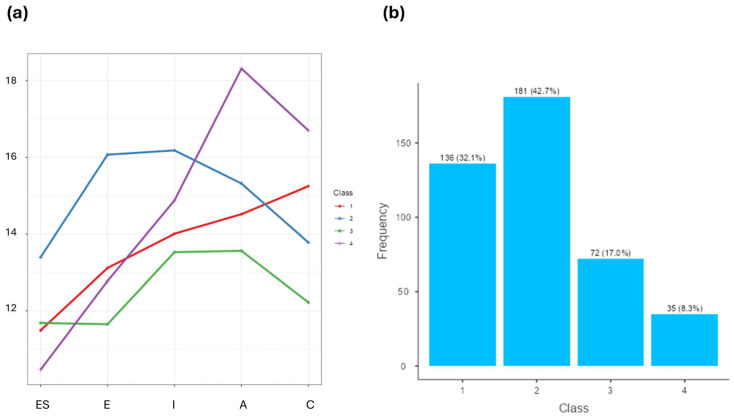
The four latent profiles identified in the athletic sample are illustrated with (**a**) mean scores in personality traits and (**b**) prevalence across the entire sample.

Assessing the classification quality in LPA involves evaluating how well the model separates individuals into distinct, homogeneous groups. Based on Average Posterior Probabilities (AvPP > 0.70 is desired), we concluded that the model classified individuals into profiles well, since AvPP was 0.82, 0.80, 0.78, and 0.82 for classes 1, 2, 3, and 4, respectively. In addition, the graphical representations showed that the classes differed significantly in their mean indicator values (Figure 1a). This suggests that class separation was appropriate. Furthermore, all profiles demonstrated sufficient case numbers (*n* > 5%), as shown in Figure 1b. LPA model stability ensures that the solution is not a local maximum and is generalizable, which means consistent findings across different random starts. The model was examined using different random starting values, and the current solution was replicated multiple times (*n* = 500). The classification (individual assignments) and parameter estimates (class means) were identical across the replications.

The line plot shows the mean scores for the four groups of athletes (latent classes) on the five personality traits: ES, E, I, A, and C (Figure 1a). We labeled the profiles as follows: *Restrained Neurotic* (Profile 1, *n* = 136, 32%), *Open Extravert* (Profile 2, *n* = 181, 42%), *Competitive Neurotic* (Profile 3, *n* = 72, 17%), and *Cooperative Perfectionist* (Profile 4, *n* = 35, 8%). Profiles 1 and 2 are the most common, while Profiles 3 and 4 are less representative (Figure 1b).

In the next steps, class membership was treated as a grouping variable in subsequent analyses (ANOVA, Pearson’s χ^2^ test of independence, hierarchical multiple linear regression). We used a one-way ANOVA to examine differences among the four profiles in personality traits and academic achievement. The Bonferroni post hoc test showed that Profile 2 (*Open Extravert*) scored significantly higher in ES than Profile 1 (*p* < 0.001), Profile 3 (*p* < 0.001), and Profile 4 (*p* < 0.001), with a large effect size, *F*(3, 420) = 26.48, *p* < 0.001, η^2^*_p_* = 0.15. Similarly, Profile 2 showed significantly higher scores for extraversion than Profile 1 (*p* < 0.001), Profile 3 (*p* < 0.001), and Profile 4 (*p* < 0.001), and the effect size was very large, *F*(3, 420) = 109.79, *p* < 0.001, η^2^*_p_* = 0.44. In addition, extraversion was significantly higher in Profile 1 than in Profile 3 (*p* < 0.001). The sample representing Profile 2 also showed significantly higher scores for Intellect than Profile 1 (*p* < 0.001), Profile 3 (*p* < 0.001), and Profile 4 (*p* < 0.001), with a large effect size, *F*(3, 420) = 54.03, *p* < 0.001, η^2^*_p_* = 0.28. Furthermore, Profile 4 scored higher on intellect than Profile 3 (*p* < 0.01). Agreeableness showed the greatest differences across all identified profiles, with a *p*-value < 0.001 and a large effect size (*F*(3, 420) = 57.36, *p* < 0.001, η^2^*_p_* = 0.29). Similarly, significant differences (*p* < 0.001) were found between conscientiousness and all other profiles, with a large effect size (F(3, 420) = 48.37, p < 0.001, η2p = 0.26). However, profiles differed slightly in terms of academic achievement, with a small effect size, *F*(3, 420) = 3.75, *p* = 0.011, η^2^*_p_* = 0.03. The post hoc test revealed that only Profile 2 had significantly higher academic achievement scores than Profile 3 (*p* = 0.016). The remaining profiles did not differ in terms of GPA.

We also conducted Pearson’s χ^2^ test of independence to examine sex differences in particular profile membership. Male athletes were more frequently members of Profiles 1, 2, and 3, whereas females were more frequently members of Profile 4 (χ^2^(3) = 22.50, *p* < 0.001, Cramer’s V = 0.23). Athletes from Poland and Ukraine were similarly represented in Profiles 1 and 3, respectively. However, Polish students prevailed in Profile 2, whereas Ukrainian students prevailed in Profile 4 (χ^2^(3) = 14.37, *p* = 0.002, Cramer’s V = 0.18).

### 3.3. Predictors of Academic Achievement Among Athletes

Hierarchical multiple linear regression was performed with academic achievement (GPA) as the dependent variable and the four LPA profiles as predictors (Table 3). Demographic variables, such as sex, study major, and country, were included as confounders in the first step of the regression model because they were found to be related to personality, as indicated by previous studies, and could confound the regression results. Model 1 was significant (*p* < 0.001) and explained 8% of the variance in academic achievement. In the second step, five personality traits, ES, E, I, A, and C, were added to Model 2, explaining an additional 9% of academic achievement (*p* < 0.001). Finally, LPA membership was included in Model 3, which significantly (*p* < 0.001) and exclusively explained an additional 2% of the variance in academic achievement, indicating that personality profiles contribute only slightly to students’ GPA in sports.

## 4. Discussion

### 4.1. Associations Between Academic Achievement and Personality Traits

This study examined the associations between the Big Five personality traits and academic achievement among sports science students in Poland and Ukraine. The hypothesis that conscientiousness and intellect are positively associated with academic achievement among athletes was confirmed in this study, consistent with previous findings (Bhattacharjee & RamKumar, 2025; Boonyapison et al., 2025; Mammadov, 2022; O’Connor & Paunonen, 2007; Stajkovic et al., 2018). Indeed, conscientiousness is the strongest predictor of academic achievement among the five personality traits, as suggested by a previous study (Stajkovic et al., 2018). Intellect (openness) consistently correlates positively with intelligence, particularly crystallized intelligence (accumulated knowledge), and research links it to broader knowledge acquisition and academic achievement (Christensen et al., 2018; DeYoung, 2014; DeYoung et al., 2012; Nusbaum & Silvia, 2011; Zimprich et al., 2009). Therefore, the present study confirms the well-established evidence of the crucial role of conscientiousness and intellect in shaping academic achievement, especially among athletes.

Although broadly similar profile patterns can be recovered across samples, our findings suggest that the *distribution* of athletes across profiles varies by country and sex. This indicates partial cross-cultural similarity in personality configurations, alongside contextual variation in profile prevalence. Accordingly, we interpret the profiles as descriptive patterns that may be comparable across contexts rather than as strictly universal typologies.

However, the correlation analysis did not reveal significant relationships among academic achievement, emotional stability, and agreeableness, which is inconsistent with previous research (Komarraju et al., 2009, 2011; Zell & Lesick, 2022). It is important to note that the students in this study showed low scores overall on emotional stability and agreeableness. Furthermore, low reliability was observed in the Mini-IPIP study, especially in the Ukrainian sample, which may be due to cultural or linguistic differences, poor translation practices, or reverse scoring, all of which may reduce internal consistency. On the other hand, such low Cronbach’s values are common in 4-item scales, and the Mini-IPIP is designed to prioritize validity over internal consistency in favor of brevity (Donnellan et al., 2006). The low reliability (Cronbach’s alpha < 0.70) of the Mini-IPIP is a documented, acceptable limitation of using ultra-brief measures, reflecting a trade-off between scale brevity and psychometric precision rather than a flaw of the instrument itself (Credé et al., 2012). In general, short scales, although effective, sacrifice internal consistency for brevity and reduced respondent fatigue. The Mini-IPIP uses only four items to measure each of the five personality traits, which inherently limits Cronbach’s alpha, a measure that increases with the number of items. The Mini-IPIP scales demonstrate acceptable criterion-related validity, predicting significant outcomes despite low internal consistency (Donnellan et al., 2006). Research indicates that alpha values for 4-item scales are often low (sometimes below 0.60), even when the scales are valid (Ponterotto & Ruckdeschel, 2007). Given its established validity in previous literature, the Mini-IPIP scales were used in this study, regarding the limitations of internal consistency in ultra-short measures (Topolewska et al., 2014).

Studies have also indicated that personality traits can change during university education, with neuroticism increasing and extraversion and conscientiousness decreasing among athletic training students, possibly due to academic stress and external factors (Hernández-Peña et al., 2022). Frequent participation in stressful events, such as sports competitions, may increase neuroticism and decrease agreeableness in athletes. Athletes are encouraged to compare and compete with one another; therefore, low agreeableness may reflect the sport’s overall competitive nature. The dynamics of personality change during academic life in a competitive environment may, at least partially, explain the current results. Another explanation is that the present findings may be affected by cultural factors and cross-generational changes. Our study also found that high extraversion is a predictor of academic achievement, although the effect was small. A meta-analysis also showed that extraversion’s effect on academic achievement was inconsistent, depending on the learning environment (Zell & Lesick, 2022). Further cross-cultural research comparing the personality traits of athletes from various countries worldwide could better explain these findings.

### 4.2. Personality Profiles Among Athletes

Previous research employing LPA to analyze the Big Five personality traits among athletes has yielded inconclusive results, revealing distinct numbers of latent classes and their interpretations (Chen et al., 2024; Mammadov et al., 2024; Nédélec et al., 2020; Rumbold et al., 2021). It is important to note that these studies incorporated various additional variables (e.g., impulsiveness, stress response, and distinct measurements of physical activity) into the LPA models, which could alter personality scores through interactions with other traits. In this study, for the first time, an LPA was conducted to examine exclusively distinct personality types among athletes. Four distinct personality types were identified, with significant differences in agreeableness and conscientiousness observed among PA students. Two opposite personality types were observed in 74% of the participants in this study. One-third of the students presented Profile 1, *Restrained Neurotic*, with low levels of all personality traits (ES, E, I, A) except conscientiousness. This type seems to demonstrate maladaptive characteristics, which are not conducive to sporting success, as well as a high risk of mental health problems and low well-being.

In contrast, Profile 2, *Open Extravert*, with particularly high extraversion, intellect, and agreeableness, was observed in 42% of athletes as the most adaptive and healthy configuration of traits, fostering well-being and both sports and academic success. Indeed, these two profiles were found to be distinctive in terms of academic achievement, as indicated by the regression model, with the Open Extravert personality type predicting better academic achievement than the *Restrained Neurotic* type. It is important to note that academic achievements among student athletes are largely related to high levels of sports performance. Two of the profiles found in this study are less frequently represented in the study sample and share relatively low emotional stability (high neuroticism) and low extraversion (members of these classes are rather introverted), moderate intellect (openness) level, and differ significantly in agreeableness and conscientiousness. We labeled Profile 3 as *Competitive Neurotic* (the lowest levels of agreeableness and conscientiousness in the entire sample) and Profile 4 as *Cooperative Perfectionist* (the highest levels of agreeableness and conscientiousness among all four personality types).

Furthermore, gender differences were found among the profile representatives, with female athlete students prevailing in Profile 4 and males more frequently represented in the remaining Profiles 1, 2, and 3. In addition, cross-cultural differences may exist, as athletes from Poland prevailed in Profile 2 and those from Ukraine in Profile 4. However, the Mini-IPIP proved less reliable among Ukrainian students than among Polish students, and the Ukrainian version has not been formally validated. A lack of knowledge about measurement invariance prevents us from conclusively determining whether differences between sexes and countries are due to measurement error or genuine cross-cultural differences.

### 4.3. Associations Between Personality Profiles and Academic Achievement in Student Athletes

Personality profiling of athletes has shown an association with academic achievement. Profile 2 members (*Open Extravert*) scored higher on academic achievement than Profile 3 members (Competitive Neurotic), according to the ANOVA results. Regression Model 3 showed that *Open Extraverts* had higher academic achievement than Restrained Neurotics (Profile 1), while controlling for sex, major specialization, country, and the Big Five personality traits as continuous variables. However, the incremental variance in GPA explained by profile membership was marginal at 2%, suggesting a significant role for variables other than Profile 2 in explaining academic achievement. Of the five personality traits, only conscientiousness, intelligence, and emotional stability were significant, and the Big Five personality model explained only 9% of the variance in academic achievement, controlling for demographic variables such as sex, major, and country.

Previous research has shown that the overall Big Five personality traits can predict long-term athletic success, with conscientiousness, extraversion, and low neuroticism emerging as the most consistent predictors across various sports and athlete populations. More successful athletes tend to score higher on conscientiousness, agreeableness, emotional stability (low neuroticism), and extraversion than less successful athletes and non-athletes, indicating that these traits support sustained performance and achievement (Piepiora & Piepiora, 2021; Steca et al., 2018; Xu & Hao, 2025). However, agreeableness and openness (intellect) have more variable effects across sports and contexts, with agreeableness sometimes being higher among more successful athletes and openness linked to exercise motivation but less consistently linked to performance (Shuai et al., 2023; Steca et al., 2018; Q. Yang et al., 2025). Extraversion influences the social aspects of sports, greater exercise motivation, and physical activity, contributing to improved sports outcomes (Piepiora, 2021; J.-H. Yang et al., 2024). In contrast, neuroticism generally shows a negative relationship with motivation and performance due to its association with emotional instability (Judge & Ilies, 2002; Q. Yang et al., 2025; Zhang, 2025). High emotional stability helps athletes manage stress and maintain their focus during competitions (Piepiora, 2021; Piepiora & Piepiora, 2021; Xu & Hao, 2025). Conscientiousness, which reflects discipline and goal orientation, is particularly critical for long-term success, as seen in elite junior handball players and adult athletes (Juhász et al., 2025; Piepiora & Piepiora, 2021). Conscientiousness is positively associated with athletic behavior and performance, often mediated by exercise self-efficacy and motivation (J.-H. Yang et al., 2024; Q. Yang et al., 2025). Research has shown that perfectionistic strivings can also enhance performance, particularly in endurance sports, by strengthening the link between anticipated and actual results (Waleriańczyk & Stolarski, 2021).

Recent meta-analytic evidence indicates that conscientiousness and extraversion have statistically significant positive correlations with sports performance, while neuroticism generally shows a negative or insignificant relationship (Shuai et al., 2023; J.-H. Yang et al., 2024). Elite athletes tend to exhibit higher conscientiousness, extraversion, agreeableness, and emotional stability (low neuroticism) than less successful athletes, suggesting that these traits support better performance outcomes (Juhász et al., 2025; Piepiora & Piepiora, 2021; Steca et al., 2018). However, differences in personality profiles also vary by sport type, with team sport champions showing lower neuroticism and higher extraversion and openness, highlighting the role of sport-specific demands (Piepiora, 2021; Piepiora & Piepiora, 2021). Some research suggests that personality differences may be more a consequence of athletic success rather than a direct cause, indicating that this relationship is complex and potentially bidirectional (Piepiora & Piepiora, 2021). Overall, personality traits contribute to elite performance but interact with other factors, such as motivation, psychological training, and sports context, to influence outcomes (Greenlees, 2020; Li et al., 2024; Shuai et al., 2023). Gender and sports type moderate the relationship between personality traits and athletic behavior and performance, with some evidence that psychological training targeting emotional balance and team communication can enhance performance (Li et al., 2024; Piepiora, 2021). Personality traits contribute to performance variability by interacting with sport-specific demands, athlete roles, and psychological factors such as motivation and mental resilience (Ayrancı & Aydin, 2025; Piepiora, 2024; Shuai et al., 2023).

### 4.4. Practical Implications of LPA Profiling

By tailoring intervention programs to align with specific LPA profiles, institutions can more effectively support student athletes in achieving both athletic and academic excellence. *Cooperative Perfectionists* may benefit from interventions that focus on resilience in the face of setbacks, such as workshops on coping with failure and perfectionism, emphasizing a growth mindset and self-compassion. These student athletes might be assigned leadership roles in group projects or team sports to leverage their cooperative strengths. Guidance on balancing high standards with self-care can help prevent overcommitment. They could also contribute to recognition programs focused on team cohesion and academic excellence.

*Competitive Neurotic* student athletes may benefit from training in social skills and cooperation. Workshops on communication, conflict resolution, and teamwork could enhance their interpersonal effectiveness. Additionally, structured academic and sports training schedules and routines may be implemented to build conscientiousness and accountability. Collaborative behaviors and academic milestones should be systematically rewarded. Counseling could address issues related to competitiveness, such as performance anxiety, frustration tolerance, and sportsmanship.

*Restrained Neurotics* may be at higher risk for maladaptive behaviors, mental health problems, and low well-being. Therefore, intervention and prevention programs for this group should focus on improving resilience and emotional regulation through workshops on coping strategies, stress management, and emotional regulation, as well as cognitive-behavioral techniques and mindfulness-based training. Sports psychologists or counselors could create peer support groups to reduce isolation and stigma. Motivational interviewing and goal-setting sessions can also help build confidence and a sense of achievement in both sports and academics. Academically successful peers or mentors can be paired with individuals characterized by the restrained neurotic type to foster positive academic habits and provide encouragement.

In particular, individuals with an adaptive personality profile can be offered leadership roles on sports teams or academic projects to further develop their strengths. They should be encouraged to mentor others, leveraging their adaptive traits to support less resilient peers. The *Adaptive* profile group should be provided with opportunities to set ambitious goals and participate in honors or advanced programs. However, sessions on balancing high achievement with well-being may be implemented to prevent burnout.

Collaboration among coaches, academic advisors, and mental health professionals ensures that interventions are personalized and holistic. Adjusted interventions are needed, and the progress of student athletes should be regularly monitored using feedback from both students and staff. Empowering adaptive and cooperative students to lead peer support and mentoring programs may significantly foster a supportive community.

### 4.5. Limitations of the Study and Future Research Directions

Although the study showed that LPA is an effective method for identifying distinct personality profiles among athletes to explain their academic achievements, it also has some limitations that prevent generalization of the results. Importantly, although several associations reached statistical significance, the overall effect sizes were modest, and the incremental contribution of profile membership beyond the trait scores was small. Therefore, these findings are best interpreted as describing meaningful but limited predictive differences in academic achievement. Although the sample size was adequate to test the study hypotheses, male athletes outnumbered female athletes. Further studies should be more balanced across sexes, including gender diversity among athletes. The cross-sectional design of this study cannot fully establish cause-and-effect relationships; therefore, future studies should use a longitudinal design. In addition, self-report questionnaires limit research primarily due to response bias and false information resulting from participants’ subjective nature, difficulty in remembering, and the desire to present themselves in a favorable light. Future studies could use experimental methods or adopt qualitative designs to address these limitations.

Although self-reported GPA is a common, cost-effective method used in research, it can be subject to systematic, non-random bias. While generally strongly correlated with actual transcripts, self-reported GPA often suffers from overreporting, in which individuals inflate their academic achievements to appear more competent or socially desirable than they actually are. Respondents may not have accurately recalled their exact cumulative GPA. Future studies can address this problem by requiring students to use official transcript verification. Ensuring that respondents know their answers are anonymous can reduce the pressure to present a socially desirable and inflated image.

The internal consistency for some Mini-IPIP subscales was lower than the desired level. This measurement imprecision may attenuate trait–GPA associations and reduce the separation and stability of the latent profiles. Lower reliability attenuates the observed correlations, potentially underestimating the true relationship between personality traits and GPA. This may lead to Type II errors, where genuine associations are missed. Imprecise measurements increase within-profile variance, making it harder to distinguish between latent profiles or classes. Low reliability can blur group boundaries and reduce the clarity and interpretability of the identified profiles. Furthermore, low reliability introduces random errors, leading to instability in class assignment across samples or time points. It undermines the replicability of latent profiles, as small measurement fluctuations can lead to different class solutions across similar datasets. Accordingly, profile interpretations should be treated as descriptive patterns rather than precise typologies, and replication using longer trait measures or latent variable approaches is needed. Because the Ukrainian version of the Mini-IPIP has not been formally validated, the results of this study should be interpreted with caution, given its Ukrainian cultural context. Future research should validate the Ukrainian version of the Mini-IPIP and verify its measurement invariance against the Polish version.

Finally, the small percentage of variance in academic achievement explained by regression analysis indicates that variables other than personality may have a greater influence. Future research should consider variables such as academic year/semester, training load, and sport type, as well as other individual-difference factors, including impulsivity, intelligence, cognitive abilities (especially attention and memory), sports abilities, procrastination, motivation, and emotional intelligence.

## 5. Conclusions

The LPA revealed four distinct personality types as a specific configuration of the Big Five traits among athletes: *Restrained Neurotic* (Profile 1), *Open Extravert* (Profile 2), *Competitive Neurotic* (Profile 3), and *Cooperative Perfectionist* (Profile 4). Most athletes (52%) were categorized as Profiles 1, 3, and 4 and identified as neurotic introverts with a moderate level of openness (intellect). Athletes with this personality trait configuration can benefit by participating in programs aimed at maintaining emotional regulation and enhancing coping skills and resilience to respond effectively to both sporting and academic challenges. In contrast, Profile 2 (42%) was characterized by high extraversion and openness, along with relatively stable emotionality (low neuroticism). Profile 2 appears to be the most adaptive in sports settings, increasing athletes’ chances of sustaining long-term well-being and success in both sports and academics.

Agreeableness and conscientiousness emerged as the most distinctive personality traits across the four profiles. Thus, we speculate that these two traits may play a crucial role in shaping athletes’ interactions with the sports environment. Profile 2 consistently showed a predictive value for academic achievement among athletes, as demonstrated by ANOVA and regression analyses. However, it is essential to note that personality explained only a small percentage of the variance in academic achievement among sports students. Further studies should address this issue by incorporating additional variables related to the social and environmental contexts of sports activities, as well as those specific to various sports disciplines, to better explain the academic achievements of student athletes. More cross-cultural research should be conducted in the future, with broader representation of sports students worldwide, to explore the role of the social environment in academic achievement among athletes.

## Figures and Tables

**Table 1:** Descriptive statistics for athletes’ personality traits and academic achievements (*N* = 424).

Variable	Range	*M*	*SD*	Skew.	Kurt.	Pearson’s *r*				
						1	2	3	4	5
1. Academic achievements	3–5	4.00	0.46	0.10	−0.07					
2. Emotional stability	4–19	12.25	2.88	0.14	0.02	−0.04				
3. Extraversion	4–20	14.01	3.19	−0.30	−0.18	0.10 *	0.18 ***			
4. Intellect	8–20	14.88	2.50	−0.11	−0.29	0.22 ***	0.16 ***	0.26 ***		
5. Agreeableness	8–20	14.93	2.43	0.01	−0.26	0.05	−0.13 **	0.12 *	0.20 ***	
6. Conscientiousness	4–20	14.08	3.10	−0.48	−0.01	0.19 ***	−0.04	−0.04	0.09	0.11 *

* *p* < 0.05, ** *p* < 0.01, *** *p* < 0.001.

**Table 2:** Overall model fit for the latent profile analysis of the Big Five personality traits (*N* = 424).

Model	Class	AIC	AWE	BIC	SABIC	CLC	KIC	Entr.
1	2	10,353.54	10,562.29	10,418.34	10,367.56	10,322.38	10,372.54	0.42
1	3	10,330.33	10,617.51	10,419.42	10,349.61	10,287.34	10,355.33	0.50
1	4	10,311.72	10,677.17	10,425.11	10,336.26	10,257.05	10,342.72	0.67
1	5	10,306.59	10,750.65	10,444.28	10,336.39	10,239.92	10,343.59	0.66

Note. AIC = Akaike’s Information Criterion, AWE = Approximate Weight of Evidence, BIC = Bayesian Information Criterion, SABIC = Sample-Size Adjusted BIC, CLC = Classification Likelihood Criterion, KIC = Kullback Information Criterion, Entr. = Entropy.

**Table 3:** Hierarchical multiple linear regression for the academic achievements of athletes (*N* = 424).

Model	Predictor	*B*	*SE*	95% CI		*t*	*p*	β	Δ*R*^2^	*R* ^2^	*F*	*df*_1_, *df*_2_	*p*
				LL	UL								
1	Intercept	3.16	0.30	2.57	3.74	10.63	<0.001		0.075	0.075	11.41	3, 420	<0.001
	Sex	−0.18	0.05	−0.27	−0.09	−3.97	<0.001	−0.39					
	Study major	0.01	0.04	−0.08	0.09	0.20	0.841	0.02					
	Country	0.22	0.04	0.13	0.30	4.92	<0.001	0.46					
2	Emotional stability	−0.02	0.01	−0.03	0.00	−2.15	0.032	−0.11	0.090	0.165	10.25	8, 415	<0.001
	Extraversion	0.00	0.01	−0.02	0.02	0.03	0.974	0.00					
	Intellect	0.03	0.01	0.01	0.05	2.84	0.005	0.15					
	Agreeableness	0.00	0.01	−0.02	0.02	−0.06	0.953	0.00					
	Conscientiousness	0.04	0.01	0.03	0.06	5.05	<0.001	0.27					
3	LPA Membership								0.018	0.183	8.38	11, 412	<0.001
	Profile 2 vs. 1	0.14	0.07	0.01	0.28	2.08	0.038	0.31					
	Profile 3 vs. 1	0.10	0.07	−0.04	0.25	1.39	0.166	0.22					
	Profile 4 vs. 1	−0.12	0.09	−0.30	0.06	−1.32	0.189	−0.26					

Note. CI = confidence interval, LL = lower level, UL = upper level. Sex is coded as female = 0, male = 1; study major is coded as Sports Sciences = 0, Physical Education Teacher = 1; country is coded as Ukraine = 0, Poland = 1.

## Data Availability

The data presented in this study are available upon request from the corresponding author.

## Abbreviations

The following abbreviations are used in this manuscript:

AHP	Analytic Hierarchy Process
AIC	Akaike’s Information Criterion
ANOVA	analysis of variance
AWE	Approximate Weight of Evidence
BIC	Bayesian Information Criterion
CLC	Classification Likelihood Criterion
GPA	grade point average
IPIP	International Personality Item Pool
KIC	Kullback Information Criterion
LPA	Latent Profile Analysis
SABIC	Sample-Size Adjusted BIC
